# Limb apraxia and the “affordance competition hypothesis”

**DOI:** 10.3389/fnhum.2015.00429

**Published:** 2015-07-28

**Authors:** Elisabeth Rounis, Glyn Humphreys

**Affiliations:** ^1^Department of Experimental Psychology, University of OxfordOxford, UK; ^2^Nuffield Department of Clinical Neurosciences, University of OxfordOxford, UK

**Keywords:** limb apraxia, ideational apraxia, ideomotor apraxia, affordance competition hypothesis, route to action model

## Abstract

Limb apraxia, a disorder of higher order motor control, has long been a challenge for clinical assessment and understanding (Leiguarda and Marsden, 2000). The deficits originally described in limb apraxia (Liepmann, 1920) have been classified by the nature of the errors made by the patients leading to, namely, ideational and ideomotor apraxia. The dual stream hypothesis (Goodale and Milner, 1992) has been used to explain these categories: ideational apraxia is thought to relate to a deficit in the concept of a movement (coded in the ventral stream). Patients have difficulty using objects, sequencing actions to interact with them or pantomiming their use. Ideomotor apraxia, on the other hand, is thought to arise from problems in the accurate implementation of movements within the dorsal stream. One of the limitations on understanding apraxia is the failure by the clinical literature to draw on knowledge of the factors determining actions in the environment. Here we emphasize the role of affordance. There is much recent work indicating that our responses to stimuli are strongly influenced by the actions that the objects “afford”, based on their physical properties and the intentions of the actor (e.g., Tucker and Ellis, 1998). The concept of affordance, originally suggested by Gibson (1979) has been incorporated in a recent model of interactive behavior that draws from findings in non-human primates, namely the “affordance competition hypothesis” (Cisek, 2007). This postulates that interactive behavior arises by a process of competition between possible actions elicited by the environment. In this paper we argue that “affordance competition” may play a role in apraxia. We review evidence that at least some aspects of apraxia may reflect an abnormal sensitivity to competition when multiple affordances are present (Riddoch et al., 1998) and/or a poor ability to exert cognitive control over this competition when it occurs. This framework suggests a new way of conceptualizing deficits in apraxia which invites further investigations in the field.

## Introduction

Limb apraxia is a heterogeneous disorder of higher order motor control affecting skilled and learnt actions. It has traditionally been classified by the nature of the errors made by patients and the brain pathways with which these errors are associated (Liepmann, 1920; Leiguarda and Marsden, 2000). Sub-aspects of the disorder have been broadly classified as reflecting impairments of the conceptual representation of actions leading to ideational apraxia, or of the implementation of these concepts, termed ideomotor apraxia, or, if the impairment pertains exclusively to skilled use of finger or hand gestures, to limb kinetic apraxia (Faglioni and Basso, 1985; Leiguarda and Marsden, 2000).

**Figure 1:**
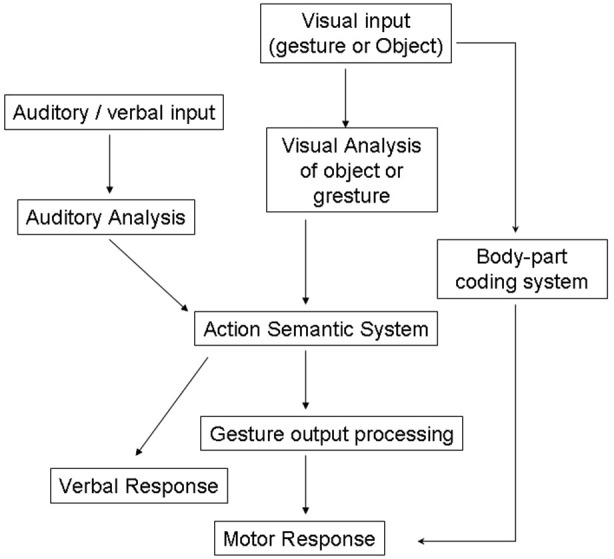
**Models of cognitive processing pathways in apraxia (Rothi et al., 1991).** Adapted from Bickerton et al. (2012) (with permission).

These definitions of apraxia have been strongly debated in the literature (Buxbaum, 2001; Buxbaum et al., 2007) but share a common basis within traditional cognitive theories which view perception, decision-making and actions serially as depicted by “box-and-arrows” models, as illustrated in Figure 1 (Heilman and Rothi, 1997).

Traditional models of apraxia have relied on observational qualitative data and they have typically remained descriptive. Sometimes they contradict observations of patients in real life leading to difficulties defining particular subtypes (e.g., ideational vs. ideomotor, Buxbaum, 2001) or the brain areas involved (e.g., parietal vs. ventral premotor, Pazzaglia et al., 2008; Kalénine et al., 2010).

This article describes a theoretical framework called the “affordance competition hypothesis” (Cisek, 2007) which offers an alternative view of apraxia. Our aim is to explore how this proposal could influence our understanding of this complex disorder.

The affordance competition hypothesis derives from ecological psychology and aims to describe real-time interactive behavior in terms of processes that specify potential actions and select between them (Cisek and Kalaska, 2010). According to this hypothesis, processes generating behavior are resolved in parallel, instead of in a serial manner, through competition between currently available opportunities and demands for action (Cisek, 2007).

We will firstly define the affordance competition hypothesis. This is followed by a review of the patient literature to identify examples that support this hypothesis. We finish by exploring predictions from this hypothesis, relevant to different aspects of limb apraxia.

## Part 1: Defining the Affordance Competition Hypothesis

Traditional cognitive theories view the selection and specification of actions in terms of an information processing framework. According to this, perception involves the construction of various levels of internal representations of the world (Biederman, 1972; Marr, 1982) that are used to inform the cognitive system which makes decisions, which in turn can be implemented into action plans by the motor system (Tversky and Kahneman, 1981; Shafir and Tversky, 1995).

Studies reported in recent neurophysiological experiments suggest that this perspective fails to provide a unified account of behavior (Cisek and Kalaska, 2005). There are cases in which functions that should be distinct and appear to involve the same regions, or even the same cells, and others in which functions that should be unified appear distributed throughout the brain. An example of the former is the role of the lateral intraparietal area (LIP). This has been proposed to include control of gaze (Snyder et al., 1997), the representation of space in a body-centered reference frame (Snyder et al., 1998), and the representation of abstract decision variables such as expected utility (Platt and Glimcher, 1999). An example of the latter derives from the neuropsychology literature, and particularly the proposal of the dual stream hypothesis (Goodale and Milner, 1992). This postulates a ventral stream pathway dedicated for object identification distinct from a dorsal stream pathway for the control of action in space, with no account of how the two may integrate to generate real-time behaviors (Schenk and McIntosh, 2010).

An alternative hypothesis for interpreting neural data, which proposes to resolve contradicting results and account for real-time interactive behaviors has been proposed (Cisek, 2007; Cisek and Kalaska, 2010). The “affordance competition hypothesis” views interactive behavior as involving simultaneous processes that specify potential motor actions and select between them.

A mathematical model by which the cerebral cortex may implement competition between representations of visually-guided reaching actions within the dorsal stream is used as an example (Cisek, 2007). In this model attended visual stimuli elicit the generation of motor plans across visuomotor regions. An action is selected to be performed by a process of competition—implemented by a neural mechanism of mutual inhibitory connections influenced by biasing inputs from decision centers.

The concept of affordance, introduced by Gibson (1979), proposes that visual objects and their properties give rise to action representations. For example a handle “affords” to be pulled. These action representations depend on contextual demands from the task (Young, 2006).

**Figure 2:**
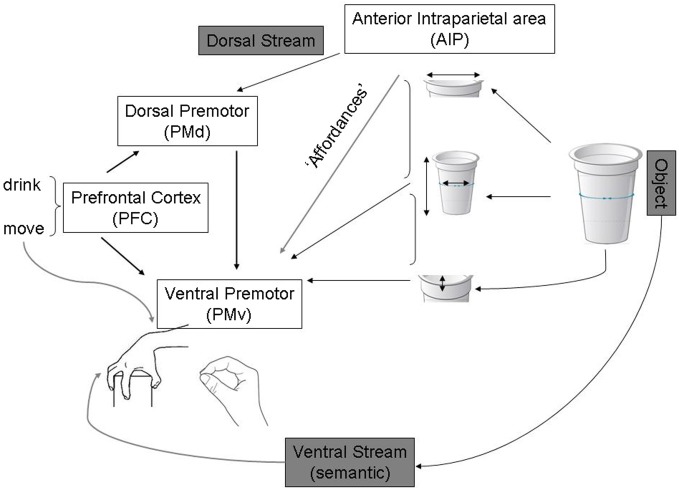
**Adapted from Fagg and Arbib (1998) publication on the Fagg-Arbib-Rizzolatti and Sakata (FARS) model for grasping.** The Anterior intraparietal area (AIP) uses visual input to extract several features of the object that are relevant to grasping it—i.e., Affordances. Ventral premotor areas represent a corresponding set of grasp options constrained by task information, instruction stimuli, working memory of recently executed grasps (represented in prefrontal areas that specify task set and influence decision making within dorsal and ventral premotor areas).

Fagg and Arbib (1998) have modelled grasp behaviors in a similar way (see Figure 2). According to this, action programming can be triggered by sensory cues without the invocation of high-level object recognition processes in the ventral stream. They emphasized the importance of current goals, tasks and internal states of the action system in determining this type of “action oriented perception” (Arbib, 1997).

Cognitive psychological studies in humans have described affordances in relation to stimulus-response compatibility effects. In a seminal experiment by Tucker and Ellis (1998), participants had to make a button press response with their right or left hand to indicate the orientation of an object presented on a screen. They observed that, when an object with an elongated handle was presented, responses were faster when the responding hand was compatible with grasping the handle relative to when the hand was in an incompatible position. Tucker and Ellis (1998) proposed that visuo-motor relations between objects and actions activate a motor response for object-use “automatically”, even when if the response is not required by the task. These compatibility effects could be elicited for different aspects of objects (e.g., the position of a handle, the size of an object) and have been referred to as “micro-affordances” (Ellis and Tucker, 2000).

However Cisek’s (2007) definition of affordances goes beyond action specification for object interactions. Rather affordances are defined as any opportunity for action provided by the environment. Cisek (2007) proposes that neural activity in the dorsal stream implements a functionally motivated mixture of variables simultaneously as sets of competing sensorimotor loops, rather than serial stages of a representation of objects in space, a representation of motor plans, or cognitive variables such as expected values. His model allows action specification to occur in parallel with action selection.

A number of predictions arise from this model which could be applicable for both abstract and object-directed actions. According to the model, each population in a neural network for action selection is proposed to involve competitive interactions, with biasing influences modulating this competition in different neural regions. Since cortico-cortical connections are bidirectional, any decision which starts to emerge in one region will propagate to other regions. In this way, decisions based on sensory features, which may be salient for action specification (see Figure 2), may first appear in parietal cortex and then influence frontal activity. In contrast decisions based on abstract rules may first be expressed in frontal regions and propagate backward to parietal areas. The competition between representations of potential actions is balanced by the accumulation of evidence in favor of a given choice leading to a decision by a process of “distributed consensus”. These proposals have implications for behaviors observed in patients, which we discuss below.

## Part 2: Examples of Affordance Competition in Patients

There are several examples in support of “affordance competition” in the animal literature (Cisek and Kalaska, 2010). In this section we review the evidence for similar processes taking place in humans, and more importantly in patient populations, relating to actions targeted to handled objects, more specific to deficits in limb apraxia. This might allow us to answer the question of whether the affordance competition hypothesis could provide a useful framework for understanding limb apraxia, going beyond previous models in the field (Rothi et al., 1991; Bartolo et al., 2001; Buxbaum et al., 2007).

Riddoch et al. (1998) studied a patient with cortico-basal degeneration who showed strong automatic grasp actions to objects. They explored a task in which the patient had to reach and grasp a cup using the hand that was on the same side of the table as the cup. When the cup was on the left, and its handle oriented to the right, the patient tended not to grasp the object with her left hand (the required response) but rather grasped it with her right hand—the action being cued by the orientation of the object in relation to the patient’s preferred hand. Interestingly this grasp action decreased when the cup was inverted, even though the physical positioning of the handle was the same as when the cup was upright. These data suggest that it was the familiar positioning of the cup, in its upright location, that triggered the grasp action to the handle. This pattern of behavior was not observed when patients were asked to point with their left or right hand depending on the location of the cup, suggesting an influence of the intended action on these affordance effects (Hommel, 2000; Linnell et al., 2005; Humphreys and Riddoch, 2007).

In a previous study, Riddoch et al. (1989) described a patient with a modality-specific deficit. This patient showed deficits in pantomiming the use of visually-presented objects only when they were asked to use their right (contralesional) hand. Patient CD had no difficulties in pantomiming actions to objects with his left hand and he was also able to pantomime actions to names using his right hand. However he had a hand- and modality-specific deficit (right hand, seen objects). The fact that the patient could pantomime actions using his right hand suggests that there was not an “ideomotor” problem in effecting right hand actions. Also the fact that he could make actions to seen objects with his left hand indicates that the problem was not an “ideational” disorder. To account for the result, Riddoch et al. (1989) proposed that CD had difficulties in selecting the appropriate action with his right hand when multiple affordances were offered by the seen object. That is, there was difficulty in selecting a hand-specific action when multiple actions were evoked for the right hand. Note that, when given the name of an object, multiple affordances would not be invoked, and CD was able to act under those conditions. These results were simulated in an explicit computational model of affordance competition by Yoon et al. (2002).

A further study reported evidence for affordance effects between multiple objects. Humphreys et al. (2000) presented patients showing utilization behavior with multiple objects and asked them to perform an unusual action with two of the items (e.g., “put the saucer on the cup”). Despite being able to repeat back the instruction, patients made errors by carrying out the familiar action (e.g., they put the cup on the saucer). This was not solely due to the familiarity of the actions offered by the objects. When asked to perform an unfamiliar action that contravened an affordance offered by the physical properties of the stimuli (e.g., “with the cup stir the pencil”—when they could make a stirring action using a cup over a pencil) patients made errors by carrying out the afforded (novel) action (e.g., stirring the pencil in the cup). Humphreys et al. (2000) proposed that affordances are offered not only by single objects but also by arrays of multiple objects which can afford different actions when used together. The affordance could be based purely on the physical properties of individual objects but also on learned interactions (as in the cup-saucer example above).The presence of multiple affordances in these more complex situations could then contribute to some of the additional symptoms associated with apraxia, such as poor sequencing of behaviors.

These pieces of evidence for both hand-specific and multi-object affordances highlight that, even when we make simple actions to objects, several affordances can be present and evoked separately for each hand and for different object combinations. In utilization behavior there is a difficulty in using task-based constraints to moderate strongly afforded actions. In apraxia there can be a problem in selecting the appropriate action when competition is present, and selection may sometimes be inappropriate leading to (amongst other things) errors in sequencing.

## Part 3: Predictions of Apraxic Deficits Based on the Affordance Competition Hypothesis

Here, we discuss some implications of the affordance competition hypothesis in relation to limb apraxia. We propose a mechanism by which models which posit a direct route to action, distinct from semantic, recognition processes, can be integrated with this framework to reflect the dynamic nature of action selection (Yoon et al., 2002).

### Action Specification and Selection Performed within Similar Networks of Brain Regions

The affordance competition hypothesis suggests that action specification and action selection are performed by the same neural circuits, distributed among a large set of brain regions.

Traditional definitions of limb apraxia have distinguished between ideational apraxia, defined as an incapacity to evoke the action associated with an object (Heilman et al., 1982), from ideomotor apraxia where patients make spatio-temporal errors in performing the appropriate gesture to a task. Ideational apraxia has also been applied to describe patients who make errors in selecting the correct target object when more than one object is present.

A major problem for the field is that the differences between these two forms of apraxia have been difficult to distinguish as few patients show one set of symptoms in isolation from symptoms characteristic of the other disorder (Buxbaum, 2001).

The affordance competition hypothesis would go further in proposing that both types of apraxia are likely to be present to some degree and that one may influence the other dynamically. In this framework ideomotor apraxia may implicate more dorsal networks for action specification, whereas more ventral networks for action selection would be related to ideational apraxia.

This parallels recent findings in the grasp literature, which have challenged the view that reach and grasp components are processed independently (Fattori et al., 2010; Vesia and Davare, 2011). Studies in non-human primates have revealed divisions within the dorsal stream (dorso-dorsal and dorso-ventral) which are thought to provide networks bridging separate functions for reach and grasp behaviors (Rizzolatti and Matelli, 2003; Daprati and Sirigu, 2006).

Similarly, tractography studies are beginning to reveal the detailed anatomical architecture of networks linking dorsal and ventral stream pathways, with direct anatomical connections between inferior parietal and temporal lobes being implicated (Heilman and Watson, 2008; Ramayya et al., 2010).

Here data from lesion mapping studies either implicating ventral premotor or inferior parietal areas in ideomotor apraxia (Haaland et al., 2000; Pazzaglia et al., 2008; Kalénine et al., 2010) may represent different facets of the same syndrome.

### Competition Leading to “Blocking” Effects and the Direct Route to Action Model

Previous neuropsychological studies describe several types of “blocking effect” in patients. For example patients with visual apraxia, who have intact object recognition and good gesturing to verbal command, may be poor at gesturing to visually presented objects (Riddoch et al., 1989). Traditional models of apraxia would predict that patients could use an intact semantic route to action (see Figure 1). However this example suggests that perceptual information interacts directly with semantic information in selecting the appropriate action to make to an object.

A convergent route model of action selection was proposed by Yoon et al. (2002) to account for this effect. They used an energy minimization network where the response derived from action selection is determined by convergent activation from separate semantic and perceptual representations. This convergent activation pushes the network into a stable state (e.g., a learned output to a given stimulus), which acts as an attractor (Hopfield, 1982). Any initial activation supplied is pushed by the dynamics of the network and by other incoming inputs into a “basin of attraction”.

In a study by Chainay and Humphreys (2002), this model was able to accurately predict behavior in a number of apraxic deficits. Most notably these authors documented apraxic action errors in a patient with impaired semantic knowledge about objects. Despite this, the use of real objects improved action—without improving semantic identification. Chainay and Humphreys (2002) argued that the sensory/perceptual input directly impinged on action specification, facilitating selection of the motor programme.

Although Yoon et al.’s (2002) model was suited for mechanisms of human action selection, recent evidence from animal neurophysiology studies have revealed that a similar process takes place in non-human primates, for specification of reaching movements (Churchland et al., 2007). The dynamics of large scale neuronal populations were decoded to generate models that account for activity in primary motor cortex (Churchland et al., 2012).

In the affordance competition hypothesis, the dynamic nature of interactions in motor responding is modelled implicitly. Indeed action representations, cued by the environment are likely available within fronto-parietal circuits, akin to the aforementioned “basins for attraction”. Biasing inputs from basal ganglia or specific cortical areas (e.g., frontal cortex, depending on the task set or parietal cortex, depending on changes in the environment) pushes the network towards a specific action by inhibiting unnecessary or competing ones.

### Subtypes of Apraxia and the Affordance Competition Hypothesis—Different Types of Affordances?

Considering apraxia under this framework reframes it as a set of disorders involving deficits in movement selection at different levels—selection of the overall movement leading to ideational apraxia or selection of specific movement parameters leading to ideomotor apraxia.

In the former case, one would predict deficits in object use arising due to there being problems in “affordances” triggering appropriate actions. This may occur because of competition between certain object characteristics which involve perception for actions, or affordances. Ideational apraxia may thus arise due to wrong actions being generated according to errors in affordances. For example, an object may be recognized for its use, yet present the actor with graspable features (affordances) that may be similar to other objects (e.g., grasping a toothbrush may be similar to grasping a knife) and lead to activation of subsequent action representations that are inappropriate for the object at hand. This maladaptive behavior may emerge from affordance triggering incorrect actions within a “state space” of action representation (cf. Chainay and Humphreys, 2002). Similarly, although the same object characteristics may generate appropriate affordances, these may in turn trigger several action representations. For example, the same object may be grasped for different uses [e.g., a pen may be grasped to write with or to move it to another location (Daprati and Sirigu, 2006)]. In this situation, patients may be unable to select from these competing actions (or inhibit them) such that an action that may be appropriate for the object but not specific to its use is performed (picking up a pen and toying with it, rather than using it to write).

In the case of ideomotor apraxia, affordance competition would predict that alternative effectors are substituted for performing an action. An example has been described by Bekkering et al. (2005). They replicated results from an original experiment reported by Goldenberg and Hagmann (1997) who used a hand and finger gesture imitation task. Meaningless gesture imitation has been used as a typical test of ideomotor apraxia because it is thought to test a “direct pathway” to gesture production that is not reliant on semantic memory or object knowledge. Bekkering et al. suggested that errors can arise in movement selection pertaining to a hierarchy of goals, with more mistakes in action selection for items lower in the action hierarchy (such as the effector used for an action) than those higher in the action hierarchy (such as action goal)—a pattern of errors also found in young children (Bekkering et al., 2000). Here, we suggest a hierarchy of goals based on a conceptual, idea-guided goal and subsequent perceptual-guided movements. Thus, when an action is observed, the action goal is observed rather than the specific movements.

Finally limb kinetic apraxia may arise because of failures in selecting the appropriate gesture or muscle configurations, from a range of possible alternatives, to perform a known and contextually relevant action (Haaland et al., 1999).

These different forms of apraxia, categorized by the affordance competition framework, may also be useful in identifying the neural correlates of the disorder(s). For example, we hypothesize that deficits in selecting appropriate actions corresponding to affordances arise from lesions to parietal cortex whereas deficits in the selection of gestures or finger movements would involve fronto-striatal circuits.

One important implication of our hypotheses is that patients with apraxia may exhibit various forms of response inhibition, due to their failure to resolve affordance competition. Studies have highlighted the importance of subcortical networks (Redgrave et al., 2010) and have identified separate top-down and bottom-up pathways in action selection (Rushworth et al., 2009; Cisek and Kalaska, 2010; Duque et al., 2012). Further studies are required to investigate whether response inhibition deficits, in their own right, contribute to apraxic deficits particularly in patient populations with basal ganglia disorders in whom apraxia has been documented (Pramstaller and Marsden, 1996; Leiguarda et al., 1997; Leiguarda and Marsden, 2000). These speculations require empirical tests.

## Conclusion

In this review, we present the affordance competition hypothesis and discuss possible implications for limb apraxia. We propose that this framework allows limb apraxia to be defined as a set of disorders in which patients are overwhelmed by the possibilities for action provided in the environment. Viewing behavior as a dynamic process in which action specification and selection occur in parallel allows for several observations to be explained such as the frequent co-existence of ideational and ideomotor deficits which have been debated at length. Moreover the framework introduces the concept of affordances as being a key trigger for action.

We believe that this framework will allow the generation of further studies through testable hypotheses that may help elucidate the complex and poorly understood disorder of apraxia.

## Conflict of Interest Statement

The authors declare that the research was conducted in the absence of any commercial or financial relationships that could be construed as a potential conflict of interest.
